# Preweaning Purified Fucoidan Drench: Effects on Growth, Immune Response, and Intestinal Morphology in Weaned Piglets

**DOI:** 10.3390/ani14101472

**Published:** 2024-05-15

**Authors:** Bonnie Homer, Reza Barekatain, Kiro R. Petrovski, Kate J. Plush, Corinna Dwan, Darryl N. D’Souza, Paul J. Verma, Roy N. Kirkwood, Bryony S. Tucker

**Affiliations:** 1 School of Animal and Veterinary Sciences, The University of Adelaide, Roseworthy, SA 5371, Australia; 2 South Australian Research and Development Institute, Roseworthy, SA 5371, Australia; 3 College of Engineering and Sciences, Flinders University, Bedford Park, SA 5042, Australia; 4 SunPork Group, Eagle Farm, QLD 4009, Australia; 5 Marinova Pty Ltd., 249 Kennedy Drive, Cambridge, TAS 7170, Australia

**Keywords:** piglets, weaning, fucoidan, gastrointestinal health, inflammation

## Abstract

**Simple Summary:**

Simple Summary: Weaning stress in piglets often leads to intestinal damage, inflammation, and subsequent problems, including diarrhea and hindered growth. While antimicrobials have been utilized to mitigate these side effects, concerns regarding antibiotic resistance necessitate the exploration of alternatives. Purified seaweed extracts, notably fucoidan, exhibit promising antioxidant and anti-inflammatory properties. This study aimed to evaluate the impact of administering a preweaning highly purified fucoidan drench to piglets on post-weaning growth, immune response, and intestinal morphology. Results demonstrated that the preweaning fucoidan drench did not elicit adverse effects on piglets. However, neither preweaning fucoidan nor an antimicrobial drench improved growth, immune response, or intestinal morphology compared to the control-treated piglets. Further research should focus on refining preweaning administrative protocols and employing commercial models to comprehensively assess fucoidan’s potential to promote the health and productivity of weaner piglets and, ultimately, reduce the industry’s reliance on antimicrobials.

**Abstract:**

Weaning stress imposes considerable physiological challenges on piglets, often manifesting in intestinal disturbances, such as inflammation and compromised barrier function, ultimately affecting growth and health outcomes. While conventional interventions, including antimicrobials, have effectively mitigated these sequelae, concerns surrounding antimicrobial resistance necessitate the exploration of alternatives. Fucoidan, derived from brown seaweed, offers promise due to its antioxidant and anti-inflammatory effects. Previous research has been limited to the in-feed supplementation of partially purified fucoidan extracted from brown seaweed. The focus of the present study is assessing the effect of a preweaning drench with highly purified (85%) fucoidan on piglet growth, immune response, and intestinal morphology post-weaning. Forty-eight male piglets at 17 ± 3 days of age (5.67 ± 0.16 kg) were assigned to a saline (control), fucoidan, or antimicrobial group, receiving treatment as a single 18 mL oral drench three days before weaning. Monitoring for seven days post-weaning included body weight measurements, blood sample collection for the inflammatory protein assay, and small intestine morphological analysis. The findings revealed that the preweaning fucoidan drench did not elicit adverse effects on piglets. However, neither fucoidan nor antimicrobial drenches significantly enhanced growth parameters, immune markers, or intestinal morphology compared to that of the control-treated piglets (*p* > 0.05). The lack of response may be attributed to the high health status of the experimental cohort and the limitation of a single dosage. Future research should consider a more challenging production setting to evaluate the viability and optimal application of fucoidan as an antimicrobial alternative in the pig industry.

## 1. Introduction

In Australian pig production, piglets are most frequently weaned between three and four weeks of age [1]. During this process, piglets encounter several challenges, including separation from the sow, changes in nutrition (composition and form) and their social and physical environment, as well as exposure to new environmental antigens and pathogens [2]. Consequently, weaning is recognized as a highly stressful period during which newly weaned piglets often experience a substantial reduction in feed intake [3,4]. This, in turn, leads to significant alterations in the morphology and function of the small intestine, manifesting as notable villous atrophy and crypt hyperplasia within two days post-weaning [4,5]. These morphological changes can significantly compromise the piglets’ ability to digest and absorb nutrients effectively [6]. In addition, the weaning process can also disrupt the intestinal barrier’s integrity, allowing foreign antigens such as bacteria, toxins, and dietary antigens to leak through the epithelium and trigger inflammation [2,7]. These intestinal changes, in combination with separation-induced stress, render newly weaned piglets more vulnerable to post-weaning diarrhea, systemic infections, and growth and productivity restrictions. These adverse outcomes, in turn, collectively contribute to significant economic losses within the pig industry [2,7,8].

To mitigate the adverse effects of weaning, prophylactic doses of antimicrobial growth promoters (AGPs) and zinc oxide (ZnO) have been employed [9]. However, due to their role in the development of antimicrobial resistance and environmental pollution, there are growing global efforts to reduce their routine incorporation in livestock diets [10]. This has prompted a search to find reliable and effective alternatives.

Seaweed extracts have emerged as promising candidates as they are considered safe, environmentally friendly, and highly bioactive [11,12,13]. One component of interest is fucoidan, a fucose-rich sulfated polysaccharide found in the cell wall of brown seaweed (*Phaeophyceae*) [14]. In the absence of in-feed AGP or ZnO, preliminary studies have shown that in-feed fucoidan supplementation extracted from a number of brown seaweed species has potential benefits for promoting health and production in weaner pigs [6,15,16,17,18]. This can be attributed to the antimicrobial, antioxidant, and anti-inflammatory properties of brown seaweeds and extracted fucoidan, which have been demonstrated in both in vitro and in vivo models [12,14,19]. In newly weaned piglets, oxidative stress often has a pivotal role in the damage inflicted on the villi and the intestinal barrier within the small intestine [4,5,7]. Fucoidan is a natural antioxidant that can scavenge free radicals and thereby minimize the extent of intestinal damage [20,21]. Additionally, anti-inflammatory activity has been shown to alleviate post-weaning inflammation [22,23]. Several studies have reported that these bioactive properties of fucoidan ultimately promote improved weaner health and growth performance [6,15,17,18]. However, there has been variation between studies regarding their experimental designs and subsequent research findings [24,25,26]. 

Some studies have shown that an antagonistic interaction exists between fucoidan and another major seaweed polysaccharide, laminarin, which may contribute to the variation observed across studies [6,16,17]. Accordingly, recent research assesses purified fucoidan extracts. However, studies have been restricted by extraction technology, resulting in the use of ‘partially purified’ or ‘fucoidan-rich’ extracts with purity levels ranging from 11% to 44% [15,24,27]. Additionally, fucoidan administration has predominantly been limited to in-feed supplementation in the weaner diet. Consequently, piglets were introduced to the supplement during periods of heightened stress and a substantial decline in feed consumption, potentially resulting in minimal fucoidan intake during the early post-weaning phase [4]. 

To date, published research assessing the effect of highly purified fucoidan in newly weaned pigs is limited, and alternative administration methods remain largely unexplored. Research involving preweaning fucoidan administration in the form of an oral drench is, to our knowledge, non-existent. Hence, the primary objective of this study was to investigate the effect of the preweaning oral dosing of a highly purified (85%) fucoidan extract from *Fucus vesiculosis* on intestinal morphology and serum inflammatory markers in weaned pigs. It was hypothesized that a preweaning fucoidan drench would reduce the extent of damage inflicted on villous structures post-weaning and thereby promote intestinal health and function as well as reduce the inflammatory response of newly weaned piglets so that they can better cope with the stressors encountered during the turbulent post-weaning period. 

## 2. Materials and Methods

This experiment was conducted at a pig production facility in Roseworthy, South Australia, from May to June 2023. The experimental protocol was approved by the Primary Industries and Regions South Australia Animal Ethics Committee (Ethics ID: #08/23). All animal procedures described were performed in agreement with the Australian Code for the Care and Use of Animals for Scientific Purposes (8th edition; National Health and Medical Research Council, 2013). 

### 2.1. Animal Housing and Management

**Figure 1:**
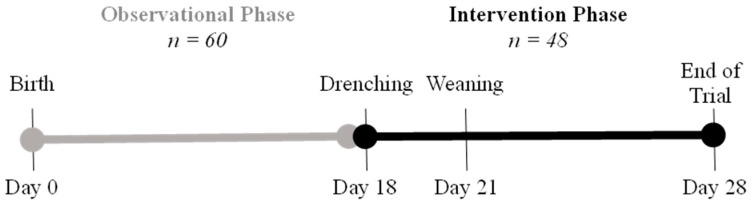
The 28-day study timeline with observational and intervention phases. The experimental day represents the average age of the intervention phase animals within a range of ±3 days.

The experiment was a 28-day study consisting of an observational phase and an intervention phase (Figure 1). The observational phase began 24 h after birth and continued through most of the lactation, during which an initial cohort of 60 male piglets were monitored. This initial cohort accounted for a potential preweaning mortality of 20% [28]. Of these, 48 seemingly healthy piglets (with no prior antimicrobial treatment) continued into the intervention phase, which commenced with a single dose of treatment administered at 18 days of age (±3 days). Blood sampling occurred on the day before treatment (day 17), at weaning (day 21), and one-week post-weaning (day 28). 

**Figure 2:**
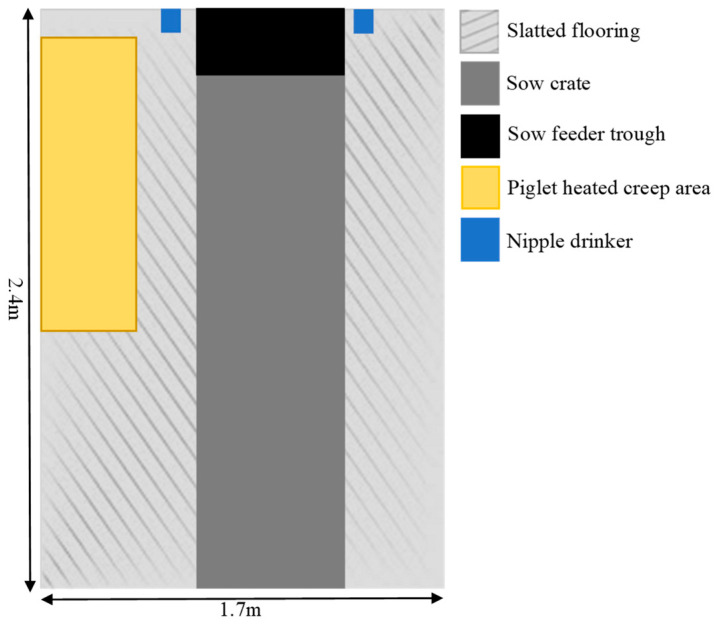
Diagram illustrating the conventional farrowing crate layout where piglets were housed with their birth sows until weaning.

During the farrowing and lactation periods, piglets were housed with their birth sow in conventional farrowing crates (1.7 × 2.4 m) with fully slatted floors and a heated creep area (Figure 2). The farrowing house was maintained at an ambient temperature of 22 °C using a thermostat-controlled cooling system. All piglet care and processing were performed by the production staff as per standard practice during the observation stage, including an iron dextran injection on day 3. At the commencement of the intervention phase (day 18), all selected piglets received 18 mL of their assigned treatment, administered orally using a 20 mL syringe. Subsequently, piglets were monitored for five minutes, and visual health checks were conducted one-hour post-treatment.

**Figure 3:**
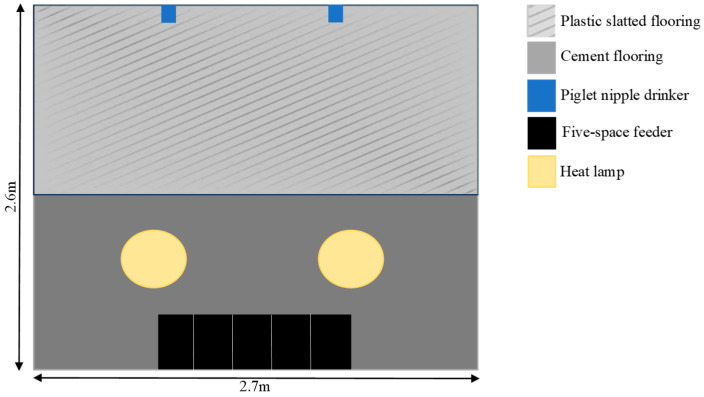
Schematic representation of the weaner pen layout where piglets were housed in their treatment groups (*n* = 16 piglets/pen) from weaning until the end of the trial.

On day 21, the piglets were weaned and relocated with their respective treatment groups from the farrowing house to three partially slatted weaning pens (2.6 × 2.7 m), each housing 16 piglets (Figure 3). Solid paneling separated the pens to minimize interactions between treatment groups. Each pen was equipped with two heat lamps. Piglets were offered unrestricted access, through a five-space feeder, to a standard commercial weaner diet formulated containing 22% crude protein, 14.5 MJ/kg of digestible energy, and 1.19% ileal digestible lysine without medication or zinc oxide inclusion. Water was available ad libitum from piglet nipple drinkers.

### 2.2. Treatment Preparation

Three treatments, including control, fucoidan, and antimicrobial, were prepared for administration as 18 mL drenches. The control treatment was 0.9% saline. The fucoidan treatment was prepared by mixing 680 mg of fucoidan with 18 mL with 0.9% saline. The fucoidan extract was obtained commercially from Marinova Pty Ltd. (Cambridge, TAS, Australia), batch no. FVS2022557. It was extracted from *Fucus vesiculosus* with a purity of 85.8% (dry weight). The chemical composition (absolute mass percentages) of the extract included 52.8% neutral carbohydrates, 22.5% sulfate, 85.8% fucoidan, and 9.5% polyphenols. The selected dosage and dilution were determined based on factors in consultation with the chemical scientist from the manufacturer. As no current research in the literature has explored the administration of brown seaweed extract as a single oral dose, the concentration was based on the optimal in-feed supplementation identified in previous studies as 240 ppm [6,16]. The single oral dose was also calculated based on the assumption of an assumed maximum daily feed intake of 0.3 kg over eight days post-weaning using an extract with 85% purity. A single dose was chosen to reduce the number of additional handling events that the piglets would experience. The antimicrobial treatment consisted of 6 mL of Scour-X^TM^ (Ausrichter Pty Ltd., Sydney, NSW, Australia), containing Sulfadiazine, Sulfadimidine, and Neomycin Sulphate, in 12 mL of 0.9% saline.

### 2.3. Experimental Design 

At approximately 24 h of age, 60 piglets were selected and ear-tagged for individual identification. Selection criteria involved sex (male) and weight (over 1 kg). Piglets from sows that were induced or medicated around the time of parturition were excluded. Selection from birth was performed to ensure the accurate tracing of birth sows and health status. To account for a potential preweaning mortality of up to 20%, the selection of 60 piglets intentionally exceeded the required 48 for the intervention phase. Based on a 5% significance level and 90% statistical power, the calculation of sample size utilized values from the literature [29] and was performed using G*Power (v3.1.9). 

Due to an outbreak of exudative epidermitis in the farrowing rooms, 26 piglets were removed from the trial at approximately day 14 as they received antibiotic treatment. To maintain a sample size with sufficient statistical power, 14 replacement piglets were selected at 17 days of age. These replacements were selected from litters previously enrolled in the trial and four additional sows. Consequently, a total of 48 piglets (body weight (BW): 5.67 ± 0.16 kg at 17 ± 3 days old) from 16 sows (Landrace × Large White; parity: 2.31 ± 0.23) progressed to the intervention phase. These 48 piglets were stratified for treatment by their litter of origin, body weight, and farrowing room. They were then subject to stratified randomization, leading to their allocation to one of the three treatment groups (*n* = 16 piglets/treatment). This allocation was performed using the standard randomization function in Microsoft Excel 365 (v2404). Subsequently, piglets received their assigned treatment drench at the outset of the intervention phase (day 18), during which samples and measurements were also collected. 

### 2.4. Experimental Measurements

#### 2.4.1. Growth Performance

Individual piglet body weights were recorded at 17, 21, and 28 days of age, and the average daily gain (ADG) was calculated. Piglets were also monitored for incidence of post-weaning diarrhea.

#### 2.4.2. Serum Sample Collection

On day 17 (noon), a baseline blood sample (5 mL) was collected from the jugular vein of each piglet into vacutainers. The samples were centrifuged (1300× *g* for 10 min at 4 °C), after which the serum was harvested and stored at −80 °C until analyses for inflammatory biomarkers were performed. This sampling procedure was repeated for all piglets at noon on day 21 (three hours after weaning) and day 28. 

#### 2.4.3. Intestinal Sample Collection

At 29 days of age, eight pigs per treatment (*n* = 24) were selected (with litter representation) and euthanized by intracardiac injection (3 mL of Lethabarb^®^, Virbac Pty Ltd., Milperra, NSW, Australia). The gastrointestinal tracts were removed within 15 min of the confirmation of death. As per Leonard et al. [30], intestinal samples (1 cm) were obtained from the duodenum (10 cm from the pylorus), the jejunum (60 cm from the pylorus), and ileum (15 cm from the caecum) and flushed with 0.9% saline. Tissue samples were then transferred to embedding cassettes and fixed in 10% neutral-buffered formalin (Sigma-Aldrich^®^ Pty Ltd., St. Louis, MO, USA) for intestinal histomorphology analysis. The tissue sample collection and analyses were performed blind for piglet identification and their allocated treatment group.

### 2.5. Laboratory Analyses

#### 2.5.1. Inflammatory Biomarkers

Assays for serum concentrations of the C reactive protein (CRP), as a major pig acute-phase protein (pig-MAP), and haptoglobin were performed using pig-specific sandwich enzyme-linked immunosorbent assay kits (Acuvet Biotech S.L., Zaragoza, Spain). These acute-phase proteins were quantified according to the manufacturer’s instructions. All analyses included the duplicate testing of standard samples and serum samples. Following serial dilutions (1:500 for CRP, 1:1000 for both pig-MAP and for haptoglobin), absorbance measurements were performed at 450 nm using a Bio-Rad xMark^TM^ Microplate Absorbance Reader. The mean absorbance values from the standard samples were used to generate a standard curve, and the best-fit line equation was employed to calculate the mean concentrations of the samples. The assays had minimum detection limits of 2 ng/mL (CRP) and 0.2 μg/mL (both pig-MAP and haptoglobin), respectively, with coefficients of variability of 6% for each.

#### 2.5.2. Intestinal Histomorphology 

**Figure 4:**
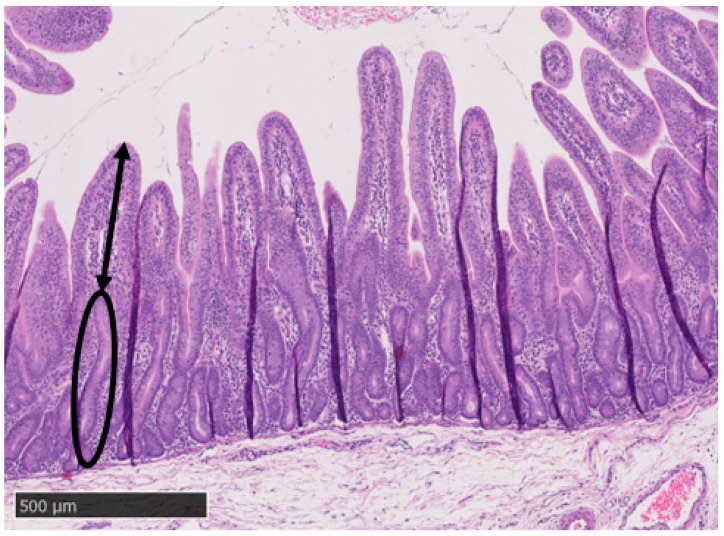
Histology image (500 µm) depicting morphometric measurements for the villous height (straight line) and the crypt depth (oval) from a jejunal tissue section (haematoxylin and eosin stained) of a piglet euthanized eight days post-weaning.

The preserved intestinal segments (duodenum, jejunum, and ileum) were prepared using standard paraffin-embedding techniques. The samples were sectioned to a thickness of 5 μm using a microtome and stained with haematoxylin and eosin. The glass slides were converted to digital slides using a Nanozoomer 2.0 Digital Slide Scanner. An image analysis program, NDP.view2 (Hamamatsu Photonics k.k., Hamamatsu, Japan), was used to measure villous height (VH) and crypt depth (CD). As per Hampson [30] and Choi et al. [15], a total of ten intact and correctly orientated villi and their associated crypts for each intestinal segment were measured. VH was measured from the crypt–villous junction to the tip of the villus, and CD was measured from the crypt–villous junction to the basement membrane (Figure 4). The results are presented as the mean VH and CD (μm), and the corresponding VH/CD ratio was calculated for each intestinal section. 

### 2.6. Statistical Analyses

The data were analyzed using the Statistical Analysis System (SAS; version 9.4; SAS Institute Inc., Cary, NC, USA). The individual piglet was the experimental unit for all dependent variables. Initially, all data were checked for normality by assessing kurtosis values using the PROC MEANS procedure (hereafter, normality is considered for kurtosis values between −2 and 2). Statistical significance was set at *p* < 0.05, while numerical trends were reported when *p* values ranged from 0.05 to 0.1. Data are presented as least-square means with standard errors of the mean.

#### 2.6.1. Growth Performance

Outliers were identified by a scatter plot and removed before further analysis (*n* = 1). The effect of treatment, farrowing room, and sow parity on the body weight (BW) and Average daily gain (ADG) of piglets were estimated using a Mixed model in PROC MIXED, as represented in Equations (1) and (2), respectively:

BW (kg) = (treatment group × farrowing room × sow parity) × e (1)

ADG (g/day) = (treatment group × farrowing room × sow parity) × e (2)

where treatment, farrowing room, and sow parity were fixed effects, and *e* represents the residual effect. Piglet age, birth litter, and BW before treatment administration were tested, but all were non-significant (*p* > 0.1) and excluded from the final model. 

#### 2.6.2. Inflammatory Biomarkers

Acute-phase inflammatory protein concentrations were assigned to normal (1) or abnormal (0) categories. Concentrations of CRP ≤ 30 μg/mL, pig-MAP ≤ 1.73 mg/mL, and haptoglobin ≤ 1.04 mg/mL were considered normal [31,32,33]. Data were then expressed as the proportion of piglets with inflammatory protein concentrations within the normal range. The effect of treatment and days since weaning on inflammatory protein levels were estimated using a regression model in PROC GLM, as represented in Equation (3):

Inflammatory Proteins = [(treatment group × day × variable) + (day × variable) + (treatment × variable)] × e(3)

where day represents the days since weaning, and the variable is the proportion of pigs within each treatment group with inflammatory protein concentrations within the normal range. These were considered as fixed effects with *e* as the residual effect. The preliminary model also tested the effects of piglet age, birth litter, farrowing room, and sow parity, but these were not significant (*p* > 0.1) and were excluded from the final model.

#### 2.6.3. Intestinal Histomorphology 

The effect of treatment, farrowing room, and sow parity on the VH, CD, and VH/CD of piglets was estimated using a Mixed model in PROC MIXED, as represented by Equation (4):

Histomorphology measure = (treatment group × farrowing room × sow parity) × e(4)

where treatment, farrowing room, and sow parity represent fixed effects, and *e* is the residual effect. The preliminary model also tested the effects of piglet age, birth litter, and body weight at day 28, but these were not significant (*p* > 0.1) and were excluded from the final model.

## 3. Results

### 3.1. Growth Performance

Preweaning drench treatments did not significantly affect piglet body weight at weaning (day 21) and seven days post-weaning (day 28). Piglet ADG was also unaffected following the treatments (*p* > 0.05; Table 1). Farrowing room and parity, however, influenced growth performance. These interactions were most pronounced in piglets originating from the farrowing room that were most severely affected by Greasy Pig Disease, where both body weight (*p* < 0.001) and ADG (*p* < 0.05) were significantly lower compared to piglets from other rooms. These farrowing rooms and parity interactions were consistent across all treatments. Additionally, no incidences of diarrhea were recorded post-weaning for any treatment group.

### 3.2. Inflammatory Biomarkers

There were no significant effects of treatment (*p* > 0.05) on acute-phase inflammatory protein concentrations at either weaning or one-week post-weaning (Table 2). A high proportion (>70%) of pigs displayed CRP and pig-MAP concentrations within the normal limits at both time points. Conversely, whilst all piglets were “within normal limits” for haptoglobin concentrations on the day of weaning, at one-week post-weaning, a large proportion (≥50%) had haptoglobin concentrations above the normal range, resulting in the mean haptoglobin concentration for the experimental cohort (1.34 mg/mL) being greater than the previously reported normal concentration (1.04 mg/mL). 

### 3.3. Intestinal Histomorphology

Fucoidan drench treatment did not affect small intestinal morphology compared to the control group (*p* > 0.05). However, compared to the piglets treated with antimicrobials, the fucoidan drench showed significantly reduced VH in the duodenum (*p* < 0.05; Table 3). No significant difference in other small intestinal measures (CD and VH/CD) was observed between these treatments. 

An interaction between farrowing rooms and small intestinal morphology was evident, particularly in piglets from the room most severely affected by exudative epidermitis. In this specific group of pigs, a significant reduction (*p* < 0.001) in VH was observed in both the duodenum (346 ± 18 μm) and jejunum (330 ± 18 μm) compared to piglets from the room least affected by the disease (duodenum: 488 ± 20 μm and jejunum: 466 ± 20 μm). This farrowing room effect was represented across the treatment groups.

## 4. Discussion

Previous studies explored the benefits of a weaner diet supplemented with brown seaweed extracts, notably fucoidan, and laminarin, on the health and performance of weaned pigs, but the findings from these studies were inconsistent. Research has previously been limited to the in-feed supplementation of ‘partially purified’ or ‘fucoidan-rich’ extracts from differing brown seaweeds with ambiguous descriptions of “purified” [15,24,27]. The focus of the present study was to assess the effect of the preweaning administration of a single dose of highly purified (85%) fucoidan extract drench on the growth, immune response, and intestinal morphology of weaned pigs.

The results indicate that the piglets treated with a preweaning fucoidan drench exhibited post-weaning intestinal morphology changes comparable to those that received the control treatment. Compared to the fucoidan drench, the antimicrobial treatment had one significant difference in morphology, with these piglets exhibiting an increase in the VH in the duodenum. This difference may be associated with differing mechanisms and sites of action, as a difference in VH was not observed in other areas of the intestine. Additionally, it did not translate into discernible production, performance, or health improvements. Neither preweaning fucoidan nor antimicrobial drench treatments significantly improved growth performance compared to the control-treated piglets. Similarly, neither treatment resulted in an enhanced immune response compared to the control treatment. 

This limited response can be primarily attributed to the overall high health status of the experimental cohort. Previous research by Pierce et al. [34] indicated that healthy and high-performing piglets often show little or no response to weaning interventions. In this study, approximately 80% of the pigs within each treatment group maintained normal, healthy levels of inflammatory CRP and pig-MAP at weaning and one-week post-weaning [31,32]. This high health status was further emphasized by the absence of post-weaning diarrhea, clinical infections and mortality, and the relatively normal growth rates among the animals in this study. Additionally, the entire experimental cohort had VH measures (a key measure of intestinal health) that consistently exceeded those observed in similar weaning fucoidan studies [6,24], where treatment-related differences in ADG, diarrhea, and intestinal morphology were noted.

Contrary to all other results indicating a cohort of high health status, the mean haptoglobin concentration of the pigs in this cohort was higher than that reported in the literature at one-week post-weaning [33,35]. This may be genetic, or it may be associated with the timing of increased levels following inflammation. In studies investigating infectious diseases in pigs, haptoglobin has a longer period of elevation compared to other acute-phase inflammatory proteins, including CRP and pig-MAP [36,37,38]. Additional serum samples throughout the seven days post-weaning, and potentially beyond, may aid in better identifying the effects of fucoidan and antimicrobials on acute-phase proteins if more gradual. 

Despite the elevated haptoglobin concentration, the overall results indicate a cohort with a high health status. While the farm’s effective management practices likely contributed to this, the weaning conditions may have played a crucial role. In contrast to the large outdoor, open shelters commonly used in Australian commercial piggeries, piglets in this trial were weaned into indoor, insulated weaning pens. In semi-outdoor settings, weaned piglets are exposed to novel pathogens, greater temperature variations, and larger groups of piglets, which can greatly challenge their immune systems [2]. Differences in immune responses between treatments might have become apparent had the piglets been exposed to the challenges of a commercial weaning environment. Despite the lack of response in the present study, there is compelling evidence suggesting that fucoidan possesses immunoregulatory and anti-inflammatory functions. In studies where piglets were challenged by lipopolysaccharide [39], or *Escherichia coli* infections [8], in-feed fucoidan supplementation was shown to enhance piglet immune responses by upregulating pro-inflammatory cytokines, including intestinal TNF-α. Additionally, fucoidan has demonstrated the ability to inhibit the attachment of bacteria, particularly pathogenic enterococci and streptococci species, in ex vivo porcine models [40]. This implies that fucoidan may exhibit immunoregulatory functions when pathogenic organisms challenge the host. Therefore, a challenge model more closely reflecting these commercial conditions could yield differing results. Research in such commercial settings is needed to explore the potential of fucoidan to control pathogens, reduce disease, and improve performance in weaned pigs.

Efforts were made to select healthy piglets not exhibiting clinical signs of exudative epidermitis. Some piglets within this study, however, were exposed to the disease before treatment during early lactation. This exposure potentially contributed to the reduced growth and intestinal morphology results observed in the piglets originating from the farrowing rooms most severely impacted by the disease. This suggests that the response to a challenging environment may be contingent on whether exposure occurs before or after drench treatment. Thus, the timing of drench administration may be important, alongside other components of the drenching protocol. 

As previous studies have been limited to in-feed fucoidan supplementation, those in the literature detailing an optimal dose, time, and duration for administering fucoidan extract as a drench are non-existent. The optimal in-feed fucoidan concentration of 240 ppm [16,18,41,42] was a reference point for establishing the drench dose in this study. The inclusion level and/or time of administration may have been insufficient to induce a notable effect when administered as a single drench. The technique of preweaning drench administration has been explored in the context of preweaning probiotics, where studies have indicated that early drench administration, specifically between birth and day four post-partum, could optimize results post-weaning [43,44,45,46]. During this early stage, the immature gut and microbiome are in a state of critical developmental plasticity, rendering them vulnerable yet receptive to changes induced by drenching interventions [47]. Furthermore, interventions targeting this developmental window have the potential to result in lasting physiological changes [48]. Other probiotic preweaning drench studies have indicated that more than one preweaning dose could be necessary to establish significant intestinal changes [45,49]. Therefore, research investigating the effects of equivalent post-partum fucoidan drench treatment is warranted. However, given the vulnerability of piglets during this early post-partum period, additional preliminary safety studies are needed.

While this study did not reveal significant differences in growth, immune response, or intestinal morphology across the treatment groups, it concluded that no adverse effects were observed when highly purified fucoidan was administered as a drench. Nevertheless, the need for further research to explore the potential efficacy of fucoidan as an antimicrobial alternative is evident. Such research is critical as, despite the increasing body of work in this area, studies have yet to consistently find suitable replacements for in-feed antimicrobials. With the growing global bans on routine antimicrobial use in livestock, identifying effective alternatives becomes even more critical. However, several challenges, including application into production, as well as concerns regarding supply, cost, and toxicity, still need to be overcome before uptake into the industry [50,51,52,53]. 

## 5. Conclusions

Piglets treated with a preweaning purified fucoidan drench exhibited comparable post-weaning growth, immune responses, and intestinal morphology to those that received an antimicrobial drench and control-treated animals. These comparable responses were likely attributed to the high health status of the experimental cohort. Further research is needed to explore the potential of purified fucoidan in challenge models reflecting commercial production systems. Moreover, additional research is required to establish the optimal drenching protocol to maximize the potential of a preweaning purified fucoidan drench to promote the health and productivity of weaning piglets and, ultimately, reduce the industry’s reliance on antimicrobials.

## Figures and Tables

**Table 1:** Mean (±standard error) piglet growth performance following preweaning drench treatments (n = 16/treatment).

	Control	Fucoidan *	Antimicrobial	*p*-Value	
				Control	Antimicrobial
BW (kg)					
d21	6.7 ± 0.3	7.1 ± 0.3	6.9 ± 0.3	0.30	0.57
d28	7.4 ± 0.3	7.6 ± 0.3	7.6 ± 0.3	0.58	0.92
ADG (g/d)					
d17–d21	246 ± 15	262 ± 15	243 ± 15	0.37	0.27
d21–d28	101 ± 14	80 ± 14	102 ± 14	0.21	0.18
d17–d28	153 ± 10	146 ± 10	153 ± 10	0.52	0.54

* Outlier removed, remaining n = 15.

**Table 2:** Mean proportions and respective 95% confidence intervals of pigs with CRP, pig-MAP, and haptoglobin concentrations within normal ranges (≤30 μg/mL, ≤1.73 mg/mL, and ≤1.04 mg/mL, respectively) on day 21 and day 28 (n = 14/treatment).

	Control	Fucoidan	Antimicrobial	*p*-Value	
				Control	Antimicrobial
CRP					
d21	0.83 (0.67–1.00)	0.82 (0.67–1.00) ^σ^	0.77 (0.60–0.97) ^β^	0.92	0.75
d28	1.00 (0.81–1.00)	0.86 (0.67–1.00)	0.79 (0.60–0.97)	0.32	0.62
Pig-MAP					
d21	0.86 (0.68–1.00)	0.86 (0.67–1.00)	0.77 (0.59–0.97)	1.00	0.69
d28	0.93 (0.74–1.00)	0.73 (0.55–0.92) ^α^	0.80 (0.62–0.98) ^α^	0.17	0.50
Haptoglobin					
d21	1.00 (0.81–1.00)	1.00 (0.81–1.00)	1.00 (0.81–1.00)	1.00	1.00
d28	0.50 (0.31–0.69)	0.36 (0.17–0.55)	0.14 (0.05–0.33)	0.29	0.11

^α, β, σ^ *n* = 15, 13, and 11, respectively, due to poor quality or insufficient quantity of blood collected.

**Table 3:** Mean (±standard error) effects of preweaning drench treatments on small intestinal VH, CD, and VH/CD of piglets on day eight post-weaning (n = 8/treatment).

	Control	Fucoidan	Antimicrobial	*p*-Value	
				Control	Antimicrobial
Duodenum					
Villous Height, μm	409 ± 31 ^a^	411 ± 33 ^a,b^	463 ± 32 ^b^	0.92	0.03
Crypt Depth, μm	457 ± 17	424 ± 15	424 ± 17	0.14	0.99
VH/CD	0.95 ± 0.09	1.02 ± 0.11	1.14 ± 0.09	1.00	1.00
Jejunum					
Villous Height, μm	393 ± 22	399 ± 33	407 ± 27	0.80	0.71
Crypt Depth, μm	381 ± 17	360 ± 10	370 ± 18	0.37	0.68
VH/CD	1.06 ± 0.03	1.14 ± 0.11	1.14 ± 0.06	1.00	1.00
Ileum					
Villous Height, μm	335 ± 31	311 ± 21	370 ± 17	0.30	0.65
Crypt Depth, μm	276 ± 9	288 ± 9	270 ± 17	0.60	0.45
VH/CD	1.25 ± 0.13	1.11 ± 0.11	1.15 ± 0.08	1.00	1.00

^a,b^ indicates significant difference between the control and antimicrobial group for duodenum villus height.

## Data Availability

Data are available upon request to the authors.

## References

[1] Dunshea F., Kerton D.K., Cranwell P.D., Campbell R., Mullan B., King R., Power G.N., Pluske J. (2003). Lifetime and post-weaning determinants of performance indices of pigs. Aust. J. Agric. Res..

[2] Campbell J.M., Crenshaw J.D., Polo J. (2013). The biological stress of early weaned piglets. J. Anim. Sci. Biotechnol..

[3] O’Doherty J.V., Venardou B., Rattigan R., Sweeney T. (2021). Feeding Marine Polysaccharides to Alleviate the Negative Effects Associated with Weaning in Pigs. Animals.

[4] Van Beers-Schreurs H.M., Nabuurs M.J., Vellenga L., Kalsbeek-van der Valk H.J., Wensing T., Breukink H.J. (1998). Weaning and the weanling diet influence the villous height and crypt depth in the small intestine of pigs and alter the concentrations of short-chain fatty acids in the large intestine and blood. J. Nutr..

[5] Hedemann M.S., Højsgaard S., Jensen B.B. (2003). Small intestinal morphology and activity of intestinal peptidases in piglets around weaning. J. Anim. Physiol. Anim. Nutr..

[6] Walsh A.M., Sweeney T., O’Shea C.J., Doyle D.N., O’Doherty J.V. (2013). Effect of dietary laminarin and fucoidan on selected microbiota, intestinal morphology and immune status of the newly weaned pig. Br. J. Nutr..

[7] Tang X., Xiong K., Fang R., Li M. (2022). Weaning stress and intestinal health of piglets: A review. Front. Immunol..

[8] Heim G., Sweeney T., O’Shea C.J., Doyle D.N., O’Doherty J.V. (2014). Effect of maternal supplementation with seaweed extracts on growth performance and aspects of gastrointestinal health of newly weaned piglets after challenge with enterotoxigenic *Escherichia coli* K88. Br. J. Nutr..

[9] Butaye P., Devriese L.A., Haesebrouck F. (2003). Antimicrobial growth promoters used in animal feed: Effects of less well known antibiotics on gram-positive bacteria. Clin. Microbiol. Rev..

[10] Manyi-Loh C., Mamphweli S., Meyer E., Okoh A. (2018). Antibiotic Use in Agriculture and Its Consequential Resistance in Environmental Sources: Potential Public Health Implications. Molecules.

[11] Luthuli S., Wu S., Cheng Y., Zheng X., Wu M., Tong H. (2019). Therapeutic Effects of Fucoidan: A Review on Recent Studies. Mar. Drugs.

[12] Mhadhebi L., Mhadhebi A., Robert J., Bouraoui A. (2014). Antioxidant, Anti-inflammatory and Antiproliferative Effects of Aqueous Extracts of Three Mediterranean Brown Seaweeds of the Genus Cystoseira. Iran. J. Pharm. Res..

[13] Corino C., Di Giancamillo A., Modina S.C., Rossi R. (2021). Prebiotic Effects of Seaweed Polysaccharides in Pigs. Animals.

[14] Michiels J., Skrivanova E., Missotten J., Ovyn A., Mrazek J., De Smet S., Dierick N. (2012). Intact brown seaweed (*Ascophyllum nodosum*) in diets of weaned piglets: Effects on performance, gut bacteria and morphology and plasma oxidative status. J. Anim. Physiol. Anim. Nutr..

[15] Choi Y., Hosseindoust A., Goel A., Lee S., Jha P.K., Kwon I.K., Chae B.-J. (2017). Effects of *Ecklonia cava* as fucoidan-rich algae on growth performance, nutrient digestibility, intestinal morphology and caecal microflora in weanling pigs. Asian-Australas. J. Anim. Sci..

[16] McDonnell P., Figat S., O’Doherty J.V. (2010). The effect of dietary laminarin and fucoidan in the diet of the weanling piglet on performance, selected faecal microbial populations and volatile fatty acid concentrations. Animal.

[17] O’Doherty J.V., McDonnell P., Figat S. (2010). The effect of dietary laminarin and fucoidan in the diet of the weanling piglet on performance and selected faecal microbial populations. Livest. Sci..

[18] Lynch M.B., Sweeney T., Callan J.J., O’Sullivan J.T., O’Doherty J.V. (2010). The effect of dietary Laminaria-derived laminarin and fucoidan on nutrient digestibility, nitrogen utilisation, intestinal microflora and volatile fatty acid concentration in pigs. J. Sci. Food Agric..

[19] Lan Y., Qin K., Wu S. (2024). The physiological activities of fucoidan and its application in animal breeding. Fish Shellfish Immunol..

[20] Zhang Q., Yu P.Z., Zhou G.F., Li Z.E., Xu Z.H. (2003). Studies on antioxidant activities of fucoidan from Laminaria japonica. Chin. Tradit. Herb. Drugs.

[21] Guo G., Yang W., Fan C., Lan R., Gao Z., Gan S., Yu H., Yin F., Wang Z. (2022). The effects of fucoidan as a dairy substitute on diarrhea rate and intestinal barrier function of the large intestine in weaned lambs. Front. Vet. Sci..

[22] Bahar B., O’Doherty J.V., Hayes M., Sweeney T., Michiels J., Skrivanova E., Missotten J., Ovyn A., Mrazek J., De Smet S. (2012). Extracts of brown seaweeds can attenuate the bacterial lipopolysaccharide-induced pro-inflammatory response in the porcine colon ex vivo. J. Anim. Sci..

[23] Venardou B., O’Doherty J.V., Maher S., Ryan M.T., Gath V., Ravindran R., Kiely C., Rajauria G., Garcia-Vaquero M., Sweeney T. (2022). Potential of a fucoidan-rich *Ascophyllum nodosum* extract to reduce Salmonella shedding and improve gastrointestinal health in weaned pigs naturally infected with Salmonella. J. Anim. Sci. Biotechnol..

[24] Rattigan R., Sweeney T., Vigors S., Thornton K., Rajauria G., O’Doherty A.J.V. (2019). The Effect of Increasing Inclusion Levels of a Fucoidan-Rich Extract Derived from *Ascophyllum nodosum* on Growth Performance and Aspects of Intestinal Health of Pigs Post-Weaning. Mar. Drugs.

[25] Heim G., Walsh A.M., Sweeney T., Doyle D.N., O’Shea C.J., Ryan M.T., O’Doherty J.V. (2014). Effect of seaweed-derived laminarin and fucoidan and zinc oxide on gut morphology, nutrient transporters, nutrient digestibility, growth performance and selected microbial populations in weaned pigs. Br. J. Nutr..

[26] Walsh A.M., Sweeney T., O’Shea C.J., Doyle D.N., O’Doherty J.V. (2013). Effect of supplementing varying inclusion levels of laminarin and fucoidan on growth performance, digestibility of diet components, selected faecal microbial populations and volatile fatty acid concentrations in weaned pigs. Anim. Feed Sci. Technol..

[27] Vigors S., O’Doherty J., Rattigan R., Sweeney T. (2021). Effect of Supplementing Seaweed Extracts to Pigs until d35 Post-Weaning on Performance and Aspects of Intestinal Health. Mar. Drugs.

[28] Muns R., Nuntapaitoon M., Tummaruk P. (2016). Non-infectious causes of pre-weaning mortality in piglets. Livest. Sci..

[29] Rymut H.E., Rund L.A., Bolt C.R., Villamil M.B., Southey B.R., Johnson R.W., Rodriguez-Zas S.L. (2021). The Combined Effect of Weaning Stress and Immune Activation during Pig Gestation on Serum Cytokine and Analyte Concentrations. Animals.

[30] Hampson D. (1986). Alteration in piglet small intestinal structure at weaning. Res. Vet. Sci..

[31] Piñeiro C., Piñeiro M., Morales J., Andrés M., Lorenzo E., Pozo M.D., Alava M.A., Lampreave F. (2009). Pig-MAP and haptoglobin concentration reference values in swine from commercial farms. Vet. J..

[32] Pomorska-Mól M., Kwit K., Markowska-Daniel I. (2012). Major Acute Phase Proteins in Pig Serum from Birth to Slaughter. J. Vet. Res..

[33] De U., Nandi S., Mukherjee R., Gaur G., Verma M.R. (2017). Identification of some plasma biomarkers associated with early weaning stress in crossbred piglets. Comp. Clin. Pathol..

[34] Pierce K., Callan J., McCarthy P., O’Doherty J. (2005). Performance of weanling pigs offered low or high lactose diets supplemented with avilamycin or inulin. Anim. Sci..

[35] Pomorska-Mól M., Markowska-Daniel I., Kwit K., Stępniewska K., Pejsak Z. (2013). C-reactive protein, haptoglobin, serum amyloid A and pig major acute phase protein response in pigs simultaneously infected with H1N1 swine influenza virus and *Pasteurella multocida*. BMC Vet. Res..

[36] Pomorska-Mól M., Kwit K., Pejsak Z., Markowska-Daniel I. (2014). Analysis of the acute-phase protein response in pigs to clinical and subclinical infection with H3N2 swine influenza virus. Influenza Other Respir. Viruses.

[37] Salamano G., Mellia E., Candiani D., Ingravalle F., Bruno R., Ru G., Doglione L. (2008). Changes in haptoglobin, C-reactive protein and pig-MAP during a housing period following long distance transport in swine. Vet. J..

[38] Asai T., Mori M., Okada M., Uruno K., Yazawa S., Shibata I. (1999). Elevated serum haptoglobin in pigs infected with porcine reproductive and respiratory syndrome virus. Vet. Immunol. Immunopathol..

[39] Leonard S.G., Sweeney T., Bahar B., O’Doherty J.V. (2012). Effect of maternal seaweed extract supplementation on suckling piglet growth, humoral immunity, selected microflora, and immune response after an ex vivo lipopolysaccharide challenge. J. Anim. Sci..

[40] Shibata H., Iimuro M., Uchiya N., Kawamori T., Nagaoka M., Ueyama S., Hashimoto S., Yokokura T., Sugimura T., Wakabayashi K. (2003). Preventive effects of Cladosiphon fucoidan against *Helicobacter pylori* infection in Mongolian gerbils. Helicobacter.

[41] Reilly P., O’Doherty J.V., Pierce K.M., Callan J.J., O’Sullivan J.T., Sweeney T. (2008). The effects of seaweed extract inclusion on gut morphology, selected intestinal microbiota, nutrient digestibility, volatile fatty acid concentrations and the immune status of the weaned pig. Animal.

[42] Gahan D.A., Lynch M.B., Callan J.J., O’Sullivan J.T., O’Doherty J.V. (2009). Performance of weanling piglets offered low-, medium- or high-lactose diets supplemented with a seaweed extract from *Laminaria* spp.. Animal.

[43] Liu C., Zhu Q., Chang J., Yin Q., Song A., Li Z., Wang E., Lu F. (2017). Effects of Lactobacillus casei and Enterococcus faecalis on growth performance, immune function and gut microbiota of suckling piglets. Arch. Anim. Nutr..

[44] Haupenthal L., Júnior J., Corrêa G., Silva B. (2020). Oral supplementation of probiotics on the performance and gut histo-morphology of suckling piglets. Ciênc. Rural.

[45] Hou C., Liu H., Zhang J., Zhang S., Yang F., Zeng X., Thacker P.A., Zhang G., Qiao S. (2015). Intestinal Microbiota Succession and Immunomodulatory Consequences after Introduction of *Lactobacillus reuteri* I5007 in Neonatal Piglets. PLoS ONE.

[46] Zeyner A., Boldt E. (2006). Effects of a probiotic Enterococcus faecium strain supplemented from birth to weaning on diarrhoea patterns and performance of piglets. J. Anim. Physiol. Anim. Nutr..

[47] Hansen L.H.B., Lauridsen C., Nielsen B., Jørgensen L., Canibe N. (2022). Impact of early inoculation of probiotics to suckling piglets on postweaning diarrhoea—A challenge study with Enterotoxigenic *E. coli* F18. Animal.

[48] Lewis M.C., Merrifield C.A., Berger B., Cloarec O., Duncker S., Mercenier A., Nicholson J.K., Holmes E., Bailey M. (2017). Early intervention with *Bifidobacterium lactis* NCC2818 modulates the host-microbe interface independent of the sustained changes induced by the neonatal environment. Sci. Rep..

[49] Wang Q., Sun Q., Qi R., Wang J., Qiu X., Liu Z., Huang J. (2019). Effects of Lactobacillus plantarum on the intestinal morphology, intestinal barrier function and microbiota composition of suckling piglets. J. Anim. Physiol. Anim. Nutr..

[50] Liu D., Van Belleghem J.D., de Vries C.R., Burgener E., Chen Q., Manasherob R., Aronson J.R., Amanatullah D.F., Tamma P.D., Suh G.A. (2021). The Safety and Toxicity of Phage Therapy: A Review of Animal and Clinical Studies. Viruses.

[51] Greco I., Molchanova N., Holmedal E., Jenssen H., Hummel B.D., Watts J.L., Håkansson J., Hansen P.R., Svenson J. (2020). Correlation between hemolytic activity, cytotoxicity and systemic in vivo toxicity of synthetic antimicrobial peptides. Sci. Rep..

[52] Villalba J.J., Costes-Thiré M., Ginane C. (2017). Phytochemicals in animal health: Diet selection and trade-offs between costs and benefits. Proc. Nutr. Soc..

[53] Rahman M.R.T., Fliss I., Biron E. (2022). Insights in the Development and Uses of Alternatives to Antibiotic Growth Promoters in Poultry and Swine Production. Antibiotics.

